# NoisyFlow: differentially private optimal transport using neural networks for secure biomedical data sharing across multiple institutions

**DOI:** 10.1093/bioinformatics/btag239

**Published:** 2026-07-07

**Authors:** Yunyang Li, Nikhil Khandekar, Skylar Wang, Varada Khanna, Julian Sanker, Mark B Gerstein

**Affiliations:** Department of Computer Science, Yale University, New Haven, CT 06511, United States; Department of Computer Science, Yale University, New Haven, CT 06511, United States; Department of Computer Science, Yale University, New Haven, CT 06511, United States; Department of Biostatistics, Yale School of Public Health, New Haven, CT 06511, United States; Department of Computer Science, Yale University, New Haven, CT 06511, United States; Department of Computer Science, Yale University, New Haven, CT 06511, United States; Program in Computational Biology and Biomedical Informatics, Yale University, New Haven, CT 06511, United States; Department of Statistics and Data Science, Yale University, New Haven, CT 06511, United States

## Abstract

**Motivation:**

Biomedical models improve when trained on data pooled across institutions, but sensitive patient records (e.g. genomics, clinical data, and medical images) are difficult to share due to privacy constraints. Moreover, data collected at different sites often have shifted distributions because of covariate differences (including batch effects), so privacy-preserving sharing alone cannot simply resolve cross-site mismatch. Methods that protect individuals while explicitly aligning distributions are needed to enable reliable multi-institutional analyses.

**Results:**

We present NoisyFlow, a three-stage differentially private framework for cross-institutional harmonization under distribution shift. In stage I, each site learns a differentially private flow-based generator of its local labeled distribution. In stage II, it learns a neural optimal transport map to a shared reference distribution. In stage III, a central server composes the released models to generate reference-aligned pseudo-data for downstream analysis without accessing raw records. Across four biomedical settings spanning single-cell genomics, histopathology, neurogenomics, and wearable sensing, NoisyFlow reduces distribution shift while preserving downstream utility under formal differential privacy guarantees.

**Availability and implementation:**

The implementation of NoisyFlow is available at https://github.com/gersteinlab/NoisyFlow.

## 1 Introduction

Recent advances in precision medicine and biomedical research have led to the aggregation of extensive patient data sets, providing substantial opportunities to elucidate disease mechanisms and develop targeted therapies (Roberts *et al.* 2020). For example, large-scale genomic analyses can identify biomarkers associated with complex diseases, while longitudinal sensor data from wearable devices can reveal previously unrecognized behavioral and physiological risk factors. However, effectively leveraging these rich data sources requires addressing two interrelated challenges. The first challenge is ensuring patient privacy while sharing sensitive medical data across institutions (Jwa and Poldrack 2022, Wang *et al.* 2023). The second challenge involves managing the heterogeneity of biomedical datasets, particularly in mitigating distribution shifts that arise when integrating populations from diverse clinical settings. Such challenges have led to pervasive data silos that fragment information and limit cross-institutional collaboration (Clayton *et al.* 2019, Bonomi *et al.* 2020, Alrefaei *et al.* 2022, Amorim *et al.* 2022, Wan *et al.* 2022). While enhanced data sharing could substantially improve machine learning model performance by exposing algorithms to larger and more diverse patient populations, privacy concerns often preclude such cooperation. These concerns are well-founded, as breaches in biomedical data privacy can potentially lead to insurance discrimination, stigmatization, or employment prejudice (Clayton *et al.* 2019, Bonomi *et al.* 2020, Alrefaei *et al.* 2022, Amorim *et al.* 2022, Wan *et al.* 2022). Consequently, models are typically trained on homogeneous datasets, which generally exhibit poor performance on out-of-distribution (OOD) populations (Zou and Schiebinger 2021). This limitation can potentially result in misdiagnoses and suboptimal treatment strategies.

### 1.1 Differential privacy in biomedical data

Addressing data-sharing challenges in the biomedical domain necessitates robust privacy-preserving approaches. Differential privacy (DP) has emerged as a prominent solution, offering mathematically rigorous guarantees that an algorithm’s output remains statistically indistinguishable whether or not a specific individual’s data is included. In essence, DP tests that no single patient’s information can be inferred from a model’s outputs, even if a malicious actor has access to auxiliary datasets or prior knowledge. This property can be particularly valuable when dealing with high-dimensional datasets such as genomics or medical imaging, where the risk of re-identification is nontrivial.

### 1.2 Distribution shifts and optimal transport

Distribution shifts are common in clinical and biomedical settings. Consider two healthcare centers—one in Boston and another in Tokyo. The Boston hospital may collect MRI scans with different imaging protocols and patient demographics compared to the Tokyo healthcare center, resulting in datasets with substantially distinct statistical and clinical properties. Even after ensuring privacy through DP-based federated learning, the combined dataset might exhibit shifts in patient ethnicity, age, or comorbidities, leading to differing underlying probability distributions. Naively merging these data could degrade model performance or obscure important subgroup-specific patterns.

A related requirement arises even when collaboration does not involve training a shared machine learning model. In many settings, institutions do not wish to release raw records and instead aim to contribute harmonized measurements in a common covariate or reference space (e.g. with site-specific effects removed), so that downstream analyses operate on comparable inputs. In low-dimensional settings, this is typically addressed via regression-based residualization or parametric batch correction. In high-dimensional settings such as single-cell profiles, residualization can be formulated as learning a transport map between probability distributions. Optimal transport provides a principled formulation of this mapping. It defines transformations that minimize distortion under a task-relevant cost, while preserving neighborhood structure and relative distances between samples in the representation space. Indeed, under certain assumptions, residualization reduces to a specific instance of an OT map. Crucially, however, the transport mechanism itself must be private. Releasing non-private maps or intermediate artifacts can leak individual-level information, whereas excessive noise can substantially degrade the utility of the harmonized data.

**Figure 1:**
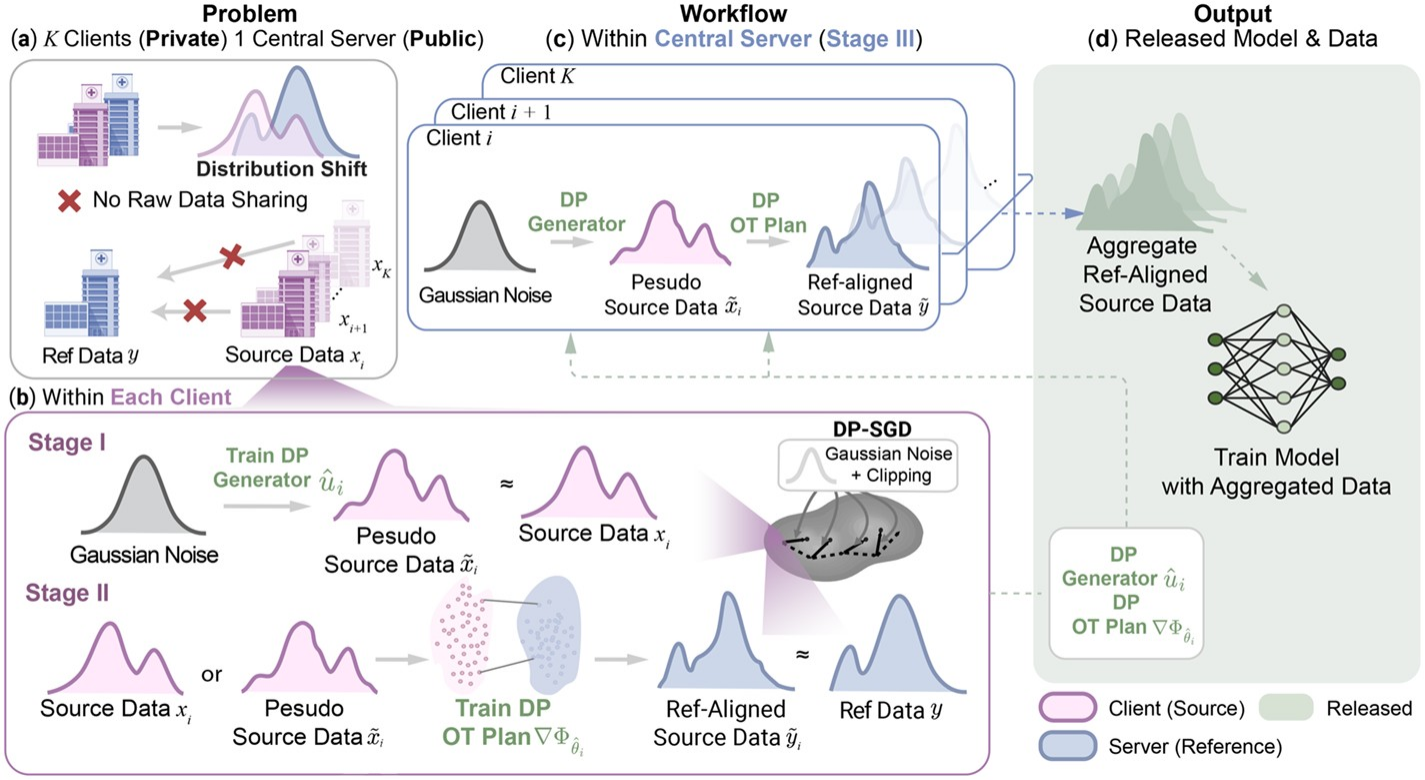
Overview of the proposed differentially private federated learning framework with optimal transport. (a) *K* clients hold private source data $x_{1},\ldots ,x_{K}$ with distribution $\mu_{s}$; a central server maintains public reference data *y* with distribution ν≠μ. No raw data is shared. (b) Each client trains a DP generator ${\hat{u}}_{i}$ (Stage I) to produce pseudo-source data ${\tilde{x}}_{i}$, and a DP optimal transport potential $\Phi_{{\hat{\theta }}_{k}}$ (Stage II) to map source data to the reference domain, yielding ${\tilde{y}}_{i}$. Both are trained via DP-SGD. (c) The central server (Stage III) uses the published DP models to generate pseudo-source data $\tilde{x}$ and maps it to the reference domain via ∇Φ(x) to obtain $\tilde{y}$. (d) The ref-aligned source data from all clients is aggregated and used to train a downstream model, which is then released along with the synthetic data, and DP-models.

Here, we present NoisyFlow, a framework that integrates differential privacy with optimal transport to support secure distribution alignment across institutions. NoisyFlow enables each client to learn DP models that (i) support sharing of a target-aligned distribution in a common reference space for privacy-preserving harmonization and downstream analyses, and (ii) support training of downstream predictive models that are less sensitive to cross-site distribution shifts. A visual overview is shown in Fig. 1.

### 1.3 Contributions

The contributions of our work can be summarized as follows: (i) We propose NoisyFlow, a differentially private optimal transport framework based on neural network parameterizations, designed for secure cross-institutional biomedical data sharing under a distribution shift. The framework supports both privacy-preserving harmonization and downstream predictive modeling without direct data exchange. (ii) We develop a practical instantiation of NoisyFlow using flow-based generative models and neural optimal transport potentials, and provide a theoretical analysis characterizing its privacy guarantees and approximation error. (iii) We conduct extensive experiments on diverse real-world biomedical datasets, including single-cell genomics, medical imaging, neurogenomics, and wearable sensor data. Results show that NoisyFlow effectively mitigates distribution shift while preserving differential privacy and improving downstream task performance, such as disease classification.

## 2 Related works

As biomedical datasets grow in complexity and volume, privacy challenges are becoming increasingly critical. Biomedical research encompasses a wide range of data modalities, including functional genomics (Gürsoy *et al.* 2022) and medical imaging, such as chest X-rays (Packhäuser *et al.* 2022). In particular, large-scale imaging datasets have driven significant advancements in cancer detection, such as for skin and breast cancer (Litjens *et al.* 2018, Vijayalakshmi 2019). However, the widespread availability and reuse of such datasets introduce substantial privacy risks, including direct genotyping from raw sequencing reads (Pajuste *et al.* 2017), inference of genotypes from expression values (Schadt *et al.* 2012), and exposure of sensitive phenotypic information through imaging analyses (Panzade *et al.* 2024). For example, expression quantitative trait loci (eQTLs) can reveal individual genotypes (Harmanci and Gerstein 2016), and imaging data might inadvertently contain identifiable health conditions. Addressing these challenges requires innovative privacy-preserving methods tailored to the multifaceted nature of biomedical data. Current approaches like privacy-preserving BAM files (Gürsoy *et al.* 2020), homomorphic encryption (Gentry 2009), and blockchain-based systems (Warnat-Herresthal *et al.* 2021) aim to balance privacy protection with data utility.

Further, recent advancements in differential privacy have led to the development of robust frameworks designed to protect biomedical data while maintaining its research utility. One key challenge in this domain is handling distribution shifts in biomedical datasets, which can affect the generalizability of models. Koh *et al.* (2021) introduced benchmarks for studying such shifts, including those present in widely used datasets like CAMELYON for breast cancer detection (Litjens *et al.* 2018). To address these challenges, LeTien *et al.* (2019) proposed a differentially private optimal transport framework for domain adaptation, demonstrating its effectiveness in mitigating distribution shifts while preserving privacy. Building on this, DP-Sinkhorn was proposed as an optimal transport-based method that avoids the instability issues commonly associated with GAN-based approaches while preserving differential privacy (Cao *et al.* 2021). This method has proven particularly effective for high-dimensional data synthesis, such as generating informative RGB images under strict privacy constraints without requiring auxiliary public data. In the realm of genomic data, specialized frameworks incorporating local differential privacy have been developed to address the unique challenges posed by correlated genetic information (Yilmaz *et al.* 2021). He *et al.* (2018) focused primarily on differential privacy for genomic data releases, highlighting limitations in handling complex genetic correlations (He *et al.* 2018). Privatedriver exemplifies a specialized privacy-preserving system that identifies cancer driver genes from multigenomics data, but it generally lacks comprehensive privacy guarantees (Song *et al.* 2024).

Optimal transport has been applied to patient-level distance detection in single-cell genomic data, offering a promising approach for privacy integration. However, it continues to face challenges in scalability and accuracy (Joodaki *et al.* 2024). Bunne *et al.* (2023) introduced CellOT, leveraging optimal transport theory within neural network architectures to map complex high-dimensional distributions.

## 3 Preliminaries and problem formulation

### 3.1 Setting and notation

We consider a federated learning scenario with *K* clients. Client i∈[K] holds a private labeled dataset $D_{i}={\left\{(x_{ij},\ell_{ij})\right\}}_{j=1}^{n_{i}},\;x_{ij}\in X\subseteq R^{d},\;\ell_{ij}\in [C],$ where X denotes the feature space (e.g. gene expression profiles or clinical measurements) and [C]={1,…,C} indexes the label set (e.g. disease subtypes or clinical outcomes). Each client’s data induces a source distribution $\mu_{i}$ over X×[C]. Without loss of generality, we assume a classification objective. Our framework extends directly to regression by replacing the discrete label set with a continuous one.

A central server holds or provides access to a *reference distribution* ν supported on $Y\subseteq R^{d}$, representing the target domain (e.g. unlabeled data from a different institution, experimental condition, or demographic cohort). (In practice, ν is usually public, but it can also be private; see Discussion.) Our goal is to train a classifier on the target domain by leveraging labeled source data from all clients, while protecting the privacy of individual records in each $D_{i}$. However, achieving this goal is complicated by the fact that source and target distributions may differ substantially. Because our framework comprises three stages with distinct privacy mechanisms, we defer the complete threat model and privacy assumptions to Section 4.5.

### 3.2 Distribution shift

A key challenge is that the source and target domains may follow different data-generating processes, resulting in distribution shift. Let μ(x) and ν(x) denote the feature distributions in the source and target domains, respectively. We distinguish two types of shift. The first arises from *implicit nuisance factors*, such as measurement protocols or acquisition conditions, which induce unobserved, domain-specific variation in *x*. The second arises from *explicit covariate effects*, where observed covariates (e.g. biological or demographic attributes) differ in distribution across domains in known ways. We next introduce optimal transport as a principled approach for aligning the resulting distributions.

### 3.3 Optimal transport

At a high level, optimal transport (OT) provides a notion of distance between probability distributions by measuring the minimal effort required to transform one distribution into another. Throughout this work, we use the quadratic cost: $c(x,y)=\frac{1}{2}∥x-y{∥}_{2}^{2},(x,y)\in X\times Y.$

Definition 3.1 (Primal Optimal Transport Problem).

*Let* μ∈P(X)*and* ν∈P(Y)*be probability measures on Polish spaces* X*and* Y*, and let* c:X×Y→[0,∞)*be a cost function. A coupling of* μ*and* ν*is a joint distribution* γ*on* X×Y*whose marginals are* μ*and* ν*; we denote the set of all such couplings by* Γ(μ,ν)*. The primal optimal transport (OT) problem seeks a coupling that minimizes the expected transportation cost:*

(1) $$\gamma^{*}\in \arg\underset{\gamma \in \Gamma (\mu ,\nu )}{\min}\int_{X\times Y}c(x,y)d\gamma (x,y).$$

*Here*, dγ(x,y)*denotes integration with respect to the joint measure* γ*over the product space* X×Y*, following standard measure-theoretic notation. Any minimizer* $\gamma^{*}$is called an optimal coupling. When $c(x,y)=∥x-y{∥}^{p}$*for* p≥1*, the optimal cost defines the p-Wasserstein distance:*

(2) $$W_{p}(\mu ,\nu ):={\left(\underset{\gamma \in \Gamma (\mu ,\nu )}{\min}\int_{X\times Y}∥x-y{∥}^{p}d\gamma (x,y)\right)}^{1/p}.$$

Intuitively, a coupling γ specifies how probability mass is transported from source locations to target locations. One can view γ as describing, for each infinitesimal amount of mass at *x*, where it should be sent in Y, subject to the constraint that the total mass sent from any region matches μ and the total mass received at any region matches ν. Many couplings satisfy these marginal constraints; optimal transport selects the one that minimizes total cost.

In practice, we work with empirical measures $\overset{⁁}{\mu }(x)=n^{-1}\sum_{i=1}^{n}\delta_{x_{i}}(x)$ and $\overset{⁁}{\nu }(y)=m^{-1}\sum_{j=1}^{m}\delta_{y_{j}}(y)$, where $\delta_{x_{i}}(x)$ denotes the Dirac delta function centered at $x_{i}$, assigning unit mass to the point $x_{i}$. In this empirical setting, the joint distribution γ reduces to a matrix $\gamma \in R_{\geq 0}^{n\times m}$, since only the finitely many sample points carry probability mass; entry $\gamma_{ij}$ indicates how much mass is sent from $x_{i}$ to $y_{j}$. The OT problem becomes a linear program: minimize $\sum_{i,j}\gamma_{ij}c(x_{i},y_{j})$ subject to the marginal constraints $\sum_{j}\gamma_{ij}=1/n$ and $\sum_{i}\gamma_{ij}=1/m$. While this primal formulation is intuitive, its dual formulation often proves more amenable to optimization.

Definition 3.2 (Kantorovich Dual). *The dual formulation of the OT problem is:*

(3) $$\underset{\varphi ,\psi }{\sup}\{\int_{X}\varphi (x)d\mu (x)+\int_{Y}\psi (y)d\nu (y)\},$$

*subject to* φ(x)+ψ(y)≤c(x,y)*for all* x∈X,y∈Y*. The functions* φ*and* ψ*are called dual potentials.*

In the discrete case, the dual is just two vectors whose sum is constrained entrywise by the cost matrix.

Under mild regularity conditions on *c*, strong duality holds and the optimal values of (1) and (3) coincide. A particularly important special case arises for the quadratic cost, where the optimal coupling is induced by a deterministic map.

Theorem 3.1 (Brenier 1991). *Let* μ*and* ν*be probability measures on* $R^{d}$*with* μ*absolutely continuous with respect to Lebesgue measure. For the quadratic cost, there exists a unique optimal transport plan* $\gamma^{*}$, and this plan is induced by a deterministic map $T^{*}:R^{d}\to R^{d}$*satisfying* $T^{*}=\nabla \Phi$*for some convex potential* $\Phi :R^{d}\to R$*. The pushforward* ${\left(T^{*}\right)}_{\#}\mu =\nu$.

The map $T^{*}$ is called the *optimal transport map* or *Monge map*. For the quadratic cost, the relationship between the dual potentials and the transport map takes a particularly elegant form.

Lemma 3.2 *Under the hypotheses of Theorem 3.1, the optimal dual potentials satisfy* $\varphi (x)=\frac{1}{2}∥x{∥}^{2}-\Phi (x)$*and* $\psi (y)=\frac{1}{2}∥y{∥}^{2}-\Phi^{*}(y)$*, where* $\Phi^{*}$denotes the convex conjugate. The optimal transport map is:

(4) $$T^{*}(x)=x-\nabla \varphi (x)=\nabla \Phi (x).$$

The results above assume access to the true distributions; in practice, we must learn the transport map from finite samples. This motivates the use of neural network approximations.

To summarize, the coupling γ represents a general *probabilistic* transport of mass (the primal solution), while the scalar potential φ characterizes the alignment through the dual formulation. The map *T* is a *deterministic* function acting on individual samples. The *pushforward* operator applies a map to a distribution, yielding the distribution of transformed samples. A coupling γ can be converted into a map T(·) via barycentric projection. Under the quadratic cost, the optimal map is recovered directly from the potential as $T^{*}(x)=x-\nabla \varphi (x)$. The goal of optimal transport is therefore to learn an optimal map T(·) that minimizes the transportation cost c(x,T(x)).

### 3.4 Differential privacy

We require that all information shared by clients satisfies differential privacy, ensuring that no adversary can infer sensitive details about individual records.

Definition 3.3 (Differential Privacy; Dwork *et al.* 2006).

*A randomized algorithm* A*is* (ε,δ)*-differentially private if for all neighboring datasets* $D∼D^{\prime }$*(differing in at most one record) and all measurable sets* S*in the output space*,

(5) $$\mathrm{Pr}[A(D)\in S]\leq e^{\varepsilon }\mathrm{Pr}[A(D^{\prime })\in S]+\delta .$$

The privacy budget ε>0 controls the privacy-utility tradeoff, with smaller values providing stronger protection. The parameter δ≥0 allows for a small probability of privacy violation, offering flexibility when exact ε-DP is infeasible.

Definition 3.4 (DP Distribution Alignment). *An alignment algorithm* $A(D_{i},D_{t})$*outputting a transport map*$T:R^{d}\to R^{d}$*is* (ε,δ)*-differentially private if, for all pairs of datasets* $D_{i}$*and* $D_{i}^{\prime }$*held by the same institution that differ in exactly one record (termed neighboring datasets), and all measurable sets* T*of transport maps*,

(6) $$\mathrm{Pr}[A(D_{i},D_{t})\in T]\leq e^{\varepsilon }\mathrm{Pr}[A(D_{i}^{\prime },D_{t})\in T]+\delta .$$

This definition ensures that the learned alignment does not reveal information about any single individual in the source dataset.

## 4 The NoisyFlow framework

NoisyFlow is a three-stage framework for differentially private domain adaptation. Each client trains a DP generative model and a DP optimal transport map; the server then synthesizes target-aligned labeled data for classifier training. We describe each stage in detail.

### 4.1 Stage I: DP flow to learn the source distribution

In order to perform OT, we require a source distribution from which to transport. A naive approach would be to directly add noise to the source data and release it. However, in high-dimensional settings, such direct perturbation severely degrades utility. To construct a usable source distribution while preserving privacy, Each client *i* learns a flow-based generative model (Lipman *et al.* 2022) with differential privacy for its source distribution. Let $\rho =N(0,I_{d})$ denote the public base distribution, a standard Gaussian from which we draw initial noise samples. We parameterize a velocity field:


(7) $${\overset{⁁}{u}}_{i}:R^{d}\times [0,1]\times [C]\to R^{d},$$

where ${\overset{⁁}{u}}_{i}$ denotes the DP-trained neural network parameters for client *i*. The velocity field takes as input a point in $R^{d}$, a time t∈[0,1], and a class label ℓ∈[C]. One could generate samples by solving the ordinary differential equation (ODE):


(8) $${\dot{z}}_{t}={\overset{⁁}{u}}_{i}(z_{t},t,\ell ),\;z_{0}∼\rho ,\;\tilde{x}:=z_{1},$$

where $z_{t}$ denotes the state at time *t*, ${\dot{z}}_{t}$ is its time derivative, $z_{0}$ is the initial noise sample, and $\tilde{x}$ is the resulting pseudo-data point. This defines a label-conditional synthetic distribution ${\overset{⁁}{\mu }}_{i}(\cdot |\ell )$, representing the learned generative model for class ℓ.

Training follows the flow matching objective. For pairs $(x,\ell )∼D_{i}$ and base samples $z_{0}∼\rho$, we define the interpolation $x_{t}=(1-t)z_{0}+tx$ for t∼Unif[0,1], which linearly interpolates between noise $z_{0}$ and data *x*. The target velocity is $u^{*}=x-z_{0}$, the direction from noise to data. The velocity is defined over one unit of time, i.e. t∈[0,1]. Client *i* minimizes:


(9) $$L_{i}^{\text{flow}}({\overset{⁁}{u}}_{i})=E_{(x,\ell )∼D_{i}}E_{z_{0}∼\rho }E_{t∼\text{Unif}[0,1]}∥{\overset{⁁}{u}}_{i}(x_{t},t,\ell )-(x-z_{0}){∥}_{2}^{2},$$

training the velocity field to match the target velocity at each interpolated point.

To ensure privacy, client *i* trains ${\overset{⁁}{u}}_{i}$ using DP-SGD (Abadi *et al.* 2016): per-example gradients are clipped to norm $C_{\text{flow}}$ (the gradient clipping threshold) and Gaussian noise with scale $\sigma_{\text{flow}}$ (the noise multiplier) is added to the minibatch gradient. The released parameters ${\overset{⁁}{u}}_{i}$ satisfy $\left(\varepsilon_{\text{flow}},\delta_{\text{flow}}\right)$-DP with respect to $D_{i}$. Here, we offer an alternative perspective in which the flow-based generative model serves as a distributional summary, analogous to privacy mechanisms that release parameterized summary statistics rather than raw data.

Optional: DP Label Prior.

If the server requires label proportions for balanced sampling, client *i* may also release a DP class prior ${\overset{⁁}{\pi }}_{i}\in \Delta^{C-1}$ by noising class counts (e.g. via the Laplace or Gaussian mechanism). Otherwise, the server may sample labels uniformly over *[C]*.

### 4.2 Stage II: DP neural OT via ICNN

In the second stage, each client learns a transport map $T_{i}:X\to Y$ that pushes source features toward the public reference distribution ν. By Theorem 3.1, for the quadratic cost $c(x,y)=\frac{1}{2}∥x-y{∥}_{2}^{2}$, the $W_{2}$-optimal Monge map takes the form T(x)=∇Φ(x) for a convex potential Φ. We parameterize $\Phi_{{\overset{⁁}{\theta }}_{i}}$ as an input-convex neural network (ICNN), where ${\overset{⁁}{\theta }}_{i}$ denotes the DP-trained neural network parameters for client *i*, and define:


(10) $$T_{{\overset{⁁}{\theta }}_{i}}(x)=\nabla_{x}\Phi_{{\overset{⁁}{\theta }}_{i}}(x).$$

Let $\Phi_{{\overset{⁁}{\theta }}_{i}}^{*}(y)=\underset{x}{\sup}\{\langle x,y\rangle -\Phi_{{\overset{⁁}{\theta }}_{i}}(x)\}$ denote the convex conjugate (Legendre transform) of $\Phi_{{\overset{⁁}{\theta }}_{i}}$. The training objective optimizes a standard convex-potential dual formulation of quadratic optimal transport:


(11) $$\underset{{\overset{⁁}{\theta }}_{i}}{\min}\;E_{x∼\mu_{i}}[\frac{1}{2}∥x{∥}_{2}^{2}-\Phi_{{\overset{⁁}{\theta }}_{i}}(x)]+E_{y∼\nu }[\frac{1}{2}∥y{∥}_{2}^{2}-\Phi_{{\overset{⁁}{\theta }}_{i}}^{*}(y)].$$

In practice, for each sampled y∼ν, we approximate $\Phi_{{\overset{⁁}{\theta }}_{i}}^{*}(y)$ by running a small number of gradient-ascent steps on *x* to solve the inner supremum. The objective is analogous to fitting an energy-based model via maximum likelihood.

To preserve the privacy, we outline three options. Under *Option A*, we optimize (11) directly on real data $x∼D_{i}$ using DP-SGD with clipping bound $C_{\text{ot}}$ and noise scale $\sigma_{\text{ot}}$, yielding $\left(\varepsilon_{\text{ot}},\delta_{\text{ot}}\right)$-DP for ${\overset{⁁}{\theta }}_{i}$. Under *Option B*, we replace real samples entirely with synthetic samples $\tilde{x}∼{\overset{⁁}{\mu }}_{i}$ generated from Stage I and train with standard (non-private) SGD; since $\tilde{x}$ derives from the DP model ${\overset{⁁}{u}}_{i}$, this constitutes post-processing and incurs no additional privacy cost. Under *Option C* (mixed training), we combine real and synthetic samples in each minibatch, applying DP-SGD only to the real-data gradient components while using standard gradients for synthetic samples. In practice, we find this can improve utility by leveraging more training signal while maintaining privacy for the real data contribution. Each client may independently select among these options based on data characteristics and privacy requirements. Once all clients have trained their models, the server can synthesize a target-aligned dataset without accessing any raw client data. We also note that in practice, Option B is preferable under a strict privacy budget because Stage II adds no privacy cost beyond Stage I; Option C typically offers the strongest utility when some additional budget is available, as it combines real-data signal with synthetic augmentation; and Option A is desirable when one prefers not to rely on generator quality.

### 4.3 Stage III: server-side aggregation

The server collects DP parameters ${\left\{({\overset{⁁}{u}}_{i},{\overset{⁁}{\theta }}_{i})\right\}}_{i=1}^{K}$ (and optionally ${\left\{{\overset{⁁}{\pi }}_{i}\right\}}_{i=1}^{K}$) and constructs a synthetic, target-aligned labeled dataset. For each client *i* and each of *M* synthetic examples, the server executes:


(12) $$\ell ∼{\overset{⁁}{\pi }}_{i},\;z∼\rho ,\;\tilde{x}=\text{Flow}({\overset{⁁}{u}}_{i};z,\ell ),\;\tilde{y}=T_{{\overset{⁁}{\theta }}_{i}}(\tilde{x}),$$

where $\text{Flow}({\overset{⁁}{u}}_{i};z,\ell )$ denotes solving the ODE (8) with initial condition *z* and label ℓ using the learned velocity field, and $\tilde{y}$ is the transported synthetic sample in the target domain. The server adds each pair $\left(\tilde{y},\ell \right)$ to the pseudo-dataset ${\tilde{D}}_{\text{DA}}$, which serves as the domain-adapted training set.

The server then trains a classifier $h_{\omega }:Y\to [C]$, parameterized by ω, by minimizing the cross-entropy loss:


(13) $$\underset{\omega }{\min}\;E_{(\tilde{y},\ell )∼{\tilde{D}}_{\text{DA}}}[\text{CE}(h_{\omega }(\tilde{y}),\ell )],$$

where CE(·,·) denotes the cross-entropy between predicted class probabilities and ground-truth labels. If unlabeled target samples from ν are available, one may optionally incorporate unsupervised regularization (e.g. entropy minimization on target predictions), though this is not required for the privacy guarantee. Having described all three stages, we now formalize the end-to-end privacy guarantee.

### 4.4 Privacy accounting

We now state the end-to-end privacy guarantee. Stage I releases ${\overset{⁁}{u}}_{i}$ with $\left(\varepsilon_{\text{flow}},\delta_{\text{flow}}\right)$-DP via DP-SGD. For Stage II: Option A releases ${\overset{⁁}{\theta }}_{i}$ with $\left(\varepsilon_{\text{ot}},\delta_{\text{ot}}\right)$-DP; Option B inherits the Stage I guarantee by post-processing with no additional cost; Option C incurs $\left(\varepsilon_{\text{ot}},\delta_{\text{ot}}\right)$-DP for the real-data component only, since synthetic gradients require no privacy budget.

Under Options A or C, sequential composition yields total client privacy:


(14) $$(\varepsilon_{i},\delta_{i})=\left(\varepsilon_{\text{flow}}+\varepsilon_{\text{ot}},\;\delta_{\text{flow}}+\delta_{\text{ot}}\right).$$

Under Option B, only the Stage I budget is consumed: $\left(\varepsilon_{i},\delta_{i})=(\varepsilon_{\text{flow}},\delta_{\text{flow}}\right)$. All server-side operations—pseudo-data generation (12) and classifier training (13)—constitute post-processing of DP-released parameters and consume no additional privacy budget. For clarity, we summarize the client and server procedures in Algorithms 1 and 2.

### 4.5 Threat model

We distinguish between private and public information in our framework. Consider a collaborative study in which multiple hospitals wish to pool knowledge for tumor classification without exposing patient records. In NoisyFlow, each hospital (client) retains its raw data locally and shares only DP-trained model parameters (${\overset{⁁}{u}}_{i}$, ${\overset{⁁}{\theta }}_{i}$, ${\overset{⁁}{\pi }}_{i}$). The coordinating server, or any other participating institution, may attempt to infer private information from these published artifacts but never observes raw records $x_{i}$, per-example gradients, or client-side random seeds. The reference distribution ν, cost function *c*, model architectures, and base noise distribution ρ (e.g. N(0,I)) are assumed public. Our privacy unit is record-level: neighboring datasets $D_{i}$ and $D_{i}^{\prime }$ differ in exactly one record.

Algorithm 1 Client *i*: DP Flow and Neural OT Training

**Require:** Private data $D_{i}$, public base ρ, public reference ν

**Ensure:** DP parameters $\left({\overset{⁁}{u}}_{i},{\overset{⁁}{\theta }}_{i}\right)$, optional ${\overset{⁁}{\pi }}_{i}$

1: **Stage I:** Train ${\overset{⁁}{u}}_{i}$ by minimizing (9) with DP-SGD (clip $C_{\text{flow}}$, noise $\sigma_{\text{flow}}$)

2: *Optional:* Compute DP label prior ${\overset{⁁}{\pi }}_{i}$ via noised counts

3: **Stage II:** Initialize ICNN potential $\Phi_{{\overset{⁁}{\theta }}_{i}}$

4: **if** Option A (real data only) **then** 

5:  Maximize (11) with $x∼D_{i}$ using DP-SGD

6: **else if** Option B (synthetic only) **then** 

7:  Sample $\tilde{x}∼{\overset{⁁}{\mu }}_{i}$ via (8); maximize (11) with standard SGD

8: **else** {Option C (mixed)}

9:  Sample $\tilde{x}∼{\overset{⁁}{\mu }}_{i}$ and $x∼D_{i}$; maximize (11) with DP-SGD on real gradients

10: **end if** 

11: Send $\left({\overset{⁁}{u}}_{i},{\overset{⁁}{\theta }}_{i},{\overset{⁁}{\pi }}_{i}\right)$ to server

Algorithm 2 Server: Transport and Aggregation

**Require:** Public reference ν, public base ρ, DP parameters ${\left\{({\overset{⁁}{u}}_{i},{\overset{⁁}{\theta }}_{i},{\overset{⁁}{\pi }}_{i})\right\}}_{i=1}^{K}$

**Ensure:** Trained classifier $h_{\overset{⁁}{\omega }}$

1: Initialize ${\tilde{D}}_{\text{DA}}\leftarrow \emptyset$

2: **for**i=1 to *K* **do** 

3:  **for**m=1 to *M* **do** 

4:   Sample $\ell ∼{\overset{⁁}{\pi }}_{i}$ (or uniform over *[C]*)

5:   Sample z∼ρ; compute $\tilde{x}=\text{Flow}({\overset{⁁}{u}}_{i};z,\ell )$

6:   Compute $\tilde{y}=\nabla \Phi_{{\overset{⁁}{\theta }}_{i}}(\tilde{x})$

7:   Add $\left(\tilde{y},\ell \right)$ to ${\tilde{D}}_{\text{DA}}$

8:  **end for** 

9: **end for** 

10: Train $h_{\omega }$ by minimizing (13) on ${\tilde{D}}_{\text{DA}}$

11: **Return**$h_{\overset{⁁}{\omega }}$

## 5 Empirical results

**Figure 2:**
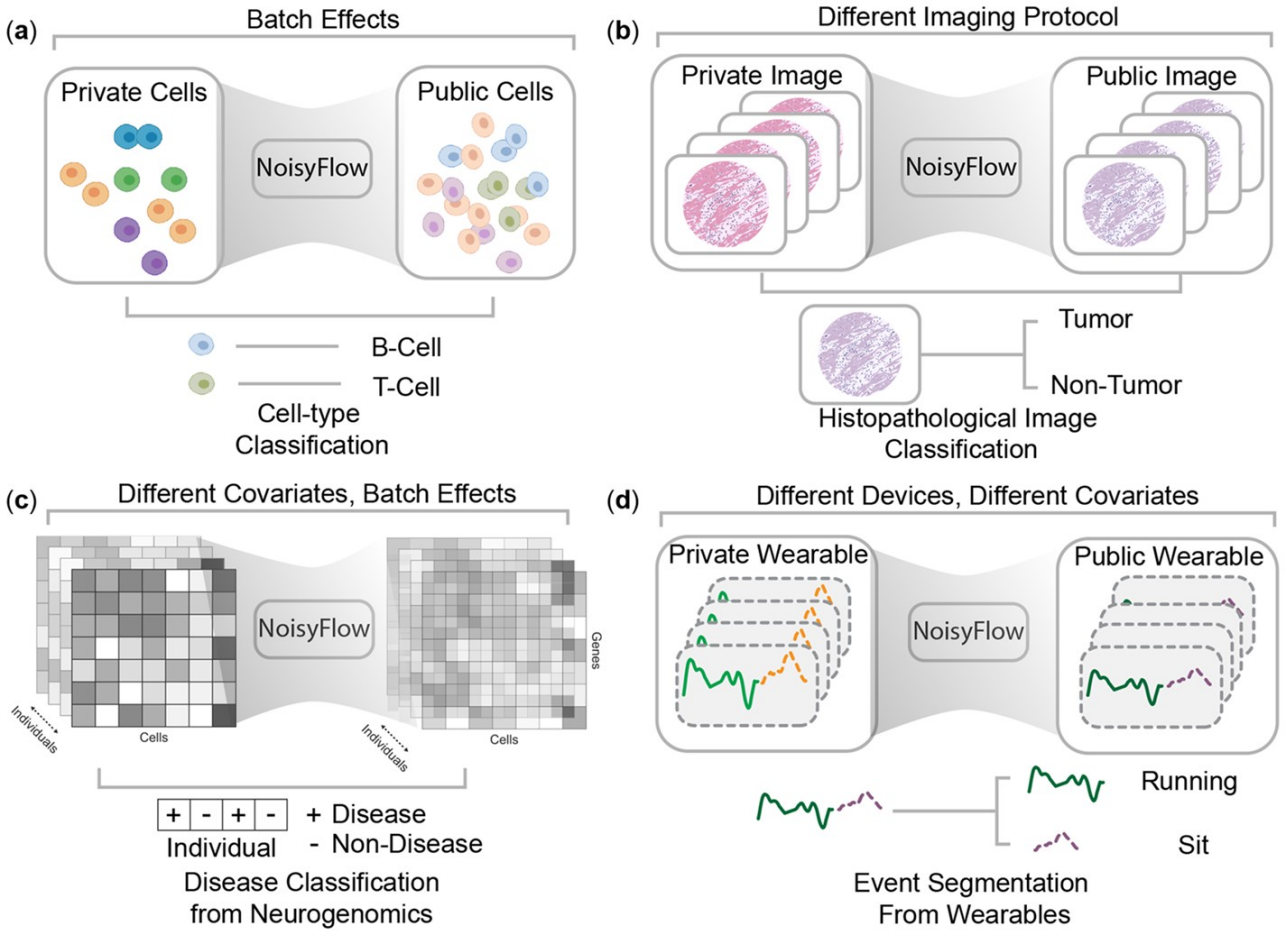
NoisyFlow enables privacy-preserving distribution alignment across biomedical modalities. Applications include (a) cell-type classification from scRNA-seq with batch effects, (b) histopathological tumor classification across imaging protocols, (c) disease classification from neurogenomics with covariate shift, and (d) activity segmentation from wearables with device heterogeneity.

Figure 2 illustrates the four biomedical application domains. We evaluate NoisyFlow on one synthetic benchmark and four real-world datasets. Utility is assessed from two complementary perspectives. First, we measure *distributional utility* by quantifying how closely the transported distribution matches the target distribution using the Sliced Wasserstein-2 (SW2) distance (Kolouri *et al.* 2019). This provides the most direct and comparable notion of utility relative to non-private optimal transport: if privacy were removed, the objective of OT is precisely to minimize the $W_{2}$ distance between source and target measures. Because the exact Wasserstein distance is computationally prohibitive in high dimensions, SW2 offers a tractable surrogate by averaging 1D Wasserstein distances over random projections. Second, we evaluate *downstream utility* through a supervised classification task, which serves as an indirect but application-relevant measure of usefulness. We first separate the reference dataset into **Ref-train** and **Ref-test**. For training the classifier, we compare three settings (i) **Ref-train-only**, trained on a limited set of labeled samples from the target distribution ν; (ii) $\tilde{y}$**-only**, trained solely on transported samples $\tilde{y}$; and (iii) **Ref-train+**$\tilde{y}$, trained on the union of real and transported data. We evaluate all the model on **Ref-test**. To isolate the contribution of the optimal transport stage, we may also train on *untransported samples* $\tilde{x}$. For the Mixture of Gaussians and single-cell datasets, we trained a random forest classifier. For CAMELYON17, we operate in a fixed embedding space using a CNN feature extractor and train an MLP on the resulting embeddings; for the wearables data, we use summary statistics computed from the time series and train a random forest on the resulting features. Improvements in downstream performance reflect whether the transported data $\tilde{y}$ provide meaningful task-level signal beyond matching marginal distributions. The empirical distributions of the ground-truth source and target are denoted by μ and ν, respectively. Unless otherwise specified, privacy guarantees are reported under (ε,δ)-differential privacy with $\delta =10^{-5}$, and ε is computed using the standard privacy accountant (Yousefpour *et al.* 2021). In federated setting, ε is computed *per client* based on that client’s local training data, and we report the worst-case privacy loss across clients. nm denotes the noise multiplier for DP-SGD.

### 5.1 Benchmark: mixture of gaussians

**Figure 3:**
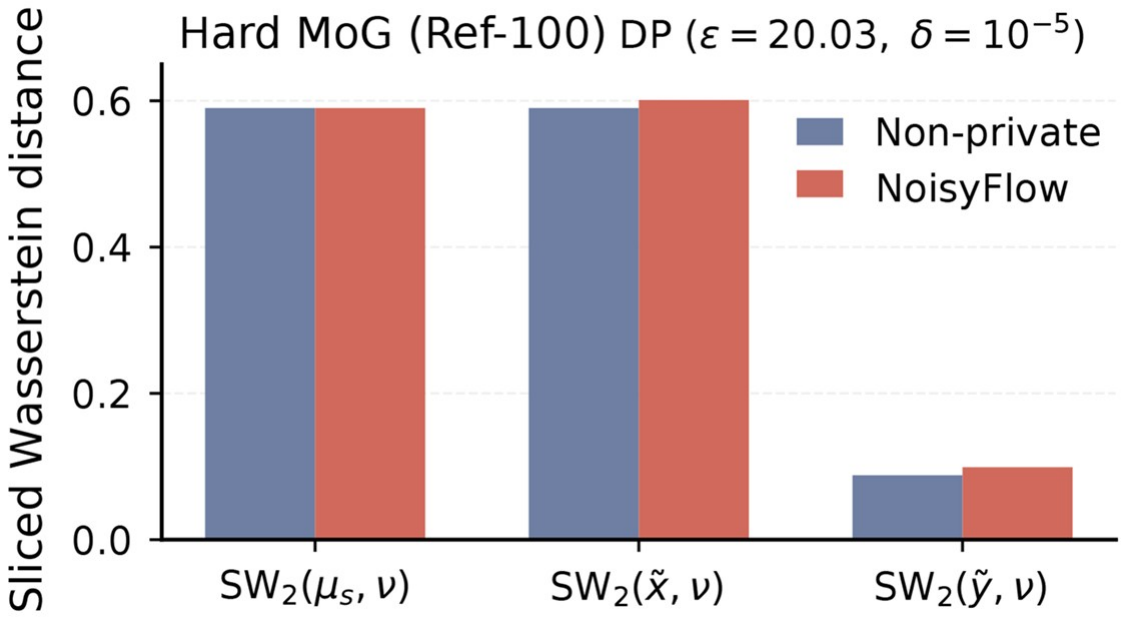
SW2 distance comparison. Raw samples $\tilde{x}$ exhibit high distance from the target, while transported samples $\tilde{y}$ achieve tight alignment under both non-private and DP settings. Results are obtained using Option C. $n_{\text{ref}}=100.$

We first validate the framework in a controlled setting where distributional shifts are known. We simulate a federated setting with K=3 clients, where each client holds data sampled from a mixture of C=6 Gaussians in $R^{50}$. To introduce covariate shift, we apply distinct random affine transformations to each client’s source distribution $\mu_{s}$. The objective is to learn a transport map $T_{\theta }$ that aligns the private client distributions to the public target ν. We tune the overlap so that the Ref-train-only baseline achieves approximately 60% target accuracy in the few-shot setting. Table 1 reports the classification accuracy of $h_{\omega }$ on the target test set. The Ref-train-only baseline achieves 60.2% accuracy. Training on transported data $\tilde{y}$ generated via $T_{\overset{⁁}{\theta }}(\tilde{x})$ yields 67.1% accuracy, confirming that the learned map successfully recovers the geometric structure of the target domain. We further quantify alignment via SW2 distance. Prior to transport, the raw samples $\tilde{x}$ exhibit a large distributional distance from the target (SW2≈0.60). Following the application of the DP OT map $T_{\overset{⁁}{\theta }}$, the distance between the transported samples $\tilde{y}$ and the target decreases to 0.099 under (ε,δ)-DP constraints (ε=20.03). This confirms that $T_{\theta }$ could effectively bridge the covariate shift while satisfying differential privacy (Fig. 3).

**Table 1:** Mixture of Gaussians benchmark.

Training data	Accuracy	Δ
Ref-train-only (y∼ν)	0.602	—
$\tilde{y}$-only ($\tilde{y}∼T_{\theta }\#\mu_{s}$)	0.671	+0.069
Ref-train+$\tilde{y}$	0.670	+0.068

Δ denotes accuracy gain over Ref-train-only. Results are obtained using Option C. $\varepsilon =20.03,\delta =10^{-5},n_{\text{ref-train}}=100$.

### 5.2 Single-cell perturbation: PBMC dataset

**Figure 4:**
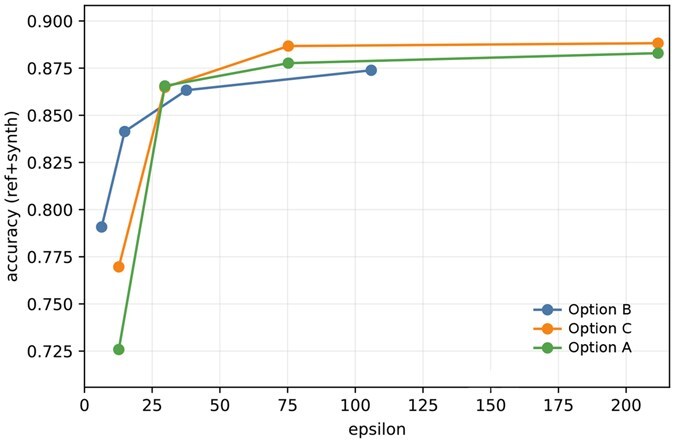
Privacy–utility trade-off. Classification accuracy as a function of the privacy budget ε when using transported samples $\tilde{y}$ for the PBMC dataset. We compare three Stage II training strategies: Option A (real-only training with DP-SGD), Option B (synthetic-only training with no additional DP cost), and Option C (mixed training on real and pseudo-data). Results are reported with $n_{\text{ref-train}}=50$, $\delta =10^{-5}$.

A central problem in immunology and drug discovery is predicting how cell fraction changes under perturbations such as drug treatment or cytokine stimulation. We use the Kang *et al.* (2018) lupus PBMC dataset, which contains 28 871 peripheral blood mononuclear cells from eight patients, profiled across 1000 highly variable genes. We formulate the task as cross-domain cell-type classification under a 2-fold domain shift. Each cell is represented by a highly variable gene expression feature vector, and the prediction target is the cell-type label. We adopt an split in which stimulated cells from patient 101 are treated as the target domain, while controlled cells from other patients serve as the private source. Stimulated cells from other patients are used for per-client optimal transport training. Performance therefore reflects both a perturbation-induced shift (control to stimulated) and an additional batch or subject shift due to evaluation on a reference patient. Table 2 presents results in a few-shot regime ($n_{\text{ref-train}}=\{5,10,50\}$). In all settings, adding transported samples $\tilde{y}$ consistently improves accuracy over Ref-train-only, with gains increasing as the number of labeled target samples $n_{\text{ref-train}}$ decreases. We also evaluate the privacy-utility trade-off. Figure 4 shows downstream accuracy as a function of the total privacy budget ε for three Stage II training options (Section 4.2). At low ε, Option B (training on raw $\tilde{x}$ only, with no DP noise in Stage II) achieves the best accuracy because it avoids additional DP noise. As ε increases, Option C (mixed training on both real source data from $D_{i}$ and raw $\tilde{x}$) surpasses both alternatives, reaching 86% accuracy at ε≈30. Option A (training on real data only with DP-SGD) converges more slowly but approaches similar performance at high ε.

**Table 2:** Few-shot cell-type classification on patient 101 in PBMC dataset.

$n_{\text{ref-train}}$	Ref-train-only	$\tilde{y}$ -only	Ref-train+$\tilde{y}$	Δ
50	0.770	0.792	0.798	+0.028
10	0.382	0.792	0.790	+0.408
5	0.258	0.792	0.793	+0.535

Comparison of Ref-train-only vs $\tilde{y}$-only vs Ref-train+$\tilde{y}$. Results are obtained with ε=9.82, using Option B. The $\tilde{y}$-only setting uses a fixed budget of 500 transported samples and does not depend on $n_{\text{ref-train}}$, so its accuracy remains constant across rows.

### 5.3 Cross-cohort neurogenomics: BrainSCOPE dataset

Large-scale neurogenomics studies aggregate transcriptomic measurements across institutions to identify molecular signatures of neurological disorders. In practice, cohort-specific experimental protocols and demographic variation induce batch effects, hindering cross-cohort model transfer. This setting directly motivates our framework: private source data from one institution must be transported to align with a target institution’s distribution.

We use a processed pseudobulk expression matrix derived from the BrainSCOPE multi-cohort snRNA-seq resource (Emani *et al.* 2024). The matrix aggregates gene expression for excitatory neurons, yielding one feature vector per individual. After filtering, we retain n=339 individuals across 12 cohorts. We define the source institution as the CMC cohort ($n_{\text{src}}=94$) and the target institution as the union of all remaining cohorts. The target is split into reference ($n_{\text{ref-train}}=196$) and test ($n_{\text{ref-test}}=49$) sets. To evaluate in a few-shot setting, we downsample the reference to $n_{\text{ref-train}}=20$.

We evaluate binary case/control classification: ℓ=0 for healthy controls, ℓ=1 for any psychiatric or neurodegenerative diagnosis (schizophrenia, bipolar disorder, ASD, Alzheimer’s, or cognitive impairment). Features are standardized and projected to d=50 principal components.

Table 3 shows that transport is necessary for cross-cohort transfer. Training on source-like synthetic samples $\tilde{x}$ without transport achieves only 61% accuracy, matching the Ref-train-only baseline with $n_{\text{ref-train}}=20$. After transport, Ref-train +$\tilde{y}$ accuracy improves to 75.5%, a gain of 14.3 percentage points. SW2 distances confirm improved alignment: transported $\tilde{y}$ (SW2 = 13.74) are notably closer to ν than raw $\tilde{x}$ (SW2 = 17.20), indicating that optimal transport recovers most of the approximation gap introduced by the DP generator. To assess the impact of the Stage I design choice, we replace the flow-based generator with a VAE on BrainSCOPE while keeping the downstream OT and classifier pipeline fixed (Table 4). While the flow-based generator performs well empirically in our setting, the framework is not tied to a specific generative model and other choices could also be used. Importantly, both variants benefit from the transport step, confirming that OT alignment drives the gain in accuracy.

**Table 3:** BrainSCOPE cross-cohort diagnosis classification.

Training data	*n*	Acc	Δ
Ref-train-only ($n_{\text{ref-train}}=20$)	20	0.612	—
$\tilde{y}$ -only	100	0.755	+0.143
Ref-train + $\tilde{y}$ (20 + 100)	120	0.755	+0.143
Ref-train + untransported $\tilde{x}$ (20 + 100)	120	0.610	−0.02

ε=9.98 , $\delta =10^{-5}$.

**Table 4:** BrainSCOPE Stage I generator ablation.

Stage I generator	Accuracy	SW2(μ,ν)	$\text{SW}2(\tilde{x},\nu )$	$\text{SW}2(\tilde{y},\nu )$
Ref-only (no generator)	0.612	—	—	—
VAE + OT	0.6367	17.20	18.35	14.85
Flow + OT	**0.755**	17.20	17.24	13.74

ε=9.98 ; 20 labeled ref points; 100 synthetic samples.

Bold values indicate the best performance in each column.

### 5.4 Histopathology: CAMELYON17-WILDS

Next, we evaluate NoisyFlow on a medical imaging task, where privacy constraints are stringent and scanner heterogeneity induces substantial distribution shift. The objective is to predict tumor presence in histopathological images. We define source and target domains at the hospital level, treating data from different hospitals as distinct distributions. In total, we consider four source hospitals and one target hospital. Table 5 reports results in a few-shot setting with $n_{\text{ref-train}}=50$.

**Table 5:** CAMELYON17-WILDS target-hospital accuracy.

Method	Private?	nm	Acc (%)	ε
*Domain adaptation baselines*				
DP-OT (MLP)	Yes	—	91.80	0.62
DP-DANN (MLP, λ=0.1)	Yes	2	91.46	0.32
*Federated baselines*				
DP-FedAvg	Yes	—	90.67	0.76
FedGP (non-private)	No	—	91.00	—
*NoisyFlow (Ours)*				
Ref-only	—	—	89.21	—
$\tilde{y}$-only	Yes	4	92.48	0.32
Ref-train+$\tilde{y}$ (Option C)	Yes	4	**92.51**	0.32

NoisyFlow outperforms domain adaptation and federated baselines under formal privacy guarantees. nm is the noise multiplier in DP-SGD. $\delta =10^{-5}$. FedGP: Jiang *et al.* (2024).

Bold values indicate the best performance in each column.

We compare against two private baselines. *DP OT* (LeTien *et al.* 2019) applies a JL transform together with additive noise to privately estimate an optimal transport plan, which is then computed using the Sinkhorn algorithm. *DP DANN* augments standard empirical risk minimization with an adversarial domain confusion objective to encourage domain-invariant representations (Ganin *et al.* 2016), with privacy enforced via DP-SGD during training. NoisyFlow with transported samples $\tilde{y}$ achieves 92.51% under ε=0.32 (Option C), outperforming both baselines, consistent with transport-based alignment being more effective than adversarial invariance in this setting. We additionally compare against predictor-only federated baselines (Table 5): DP-FedAvg reaches 90.67%, while FedGP (Jiang *et al.* 2024), a recent federated domain adaptation method using gradient projection, achieves 91.00% without any privacy constraint. NoisyFlow outperforms both at 92.51% with ε=0.32.

Table 6 provides distributional validation. Raw samples $\tilde{x}$, which approximate $\mu_{s}$, have SW2 distance ≈1.06 to the target. Applying the OT map $T_{\theta }$ reduces this to ≈0.23 for transported samples $\tilde{y}$, indicating that the ICNN potential $\Phi_{\theta }$ aligns the image embedding distribution toward ν.

**Table 6:** SW2 distance to target reference ν for the CAMELYON17-WILDS dataset.

Training scheme	nm	SW2($\mu_{s}$, ν)	SW2($\tilde{x}$, ν)	SW2($\tilde{y}$, ν)
Non-private	–	0.868	1.042	0.200
NoisyFlow (Option B)	2	0.868	1.062	0.233
NoisyFlow (Option B)	3	0.868	1.094	0.234
NoisyFlow (Option C)	4	0.868	1.097	0.223

nm is the noise multiplier in DP-SGD.

**Figure 5:**
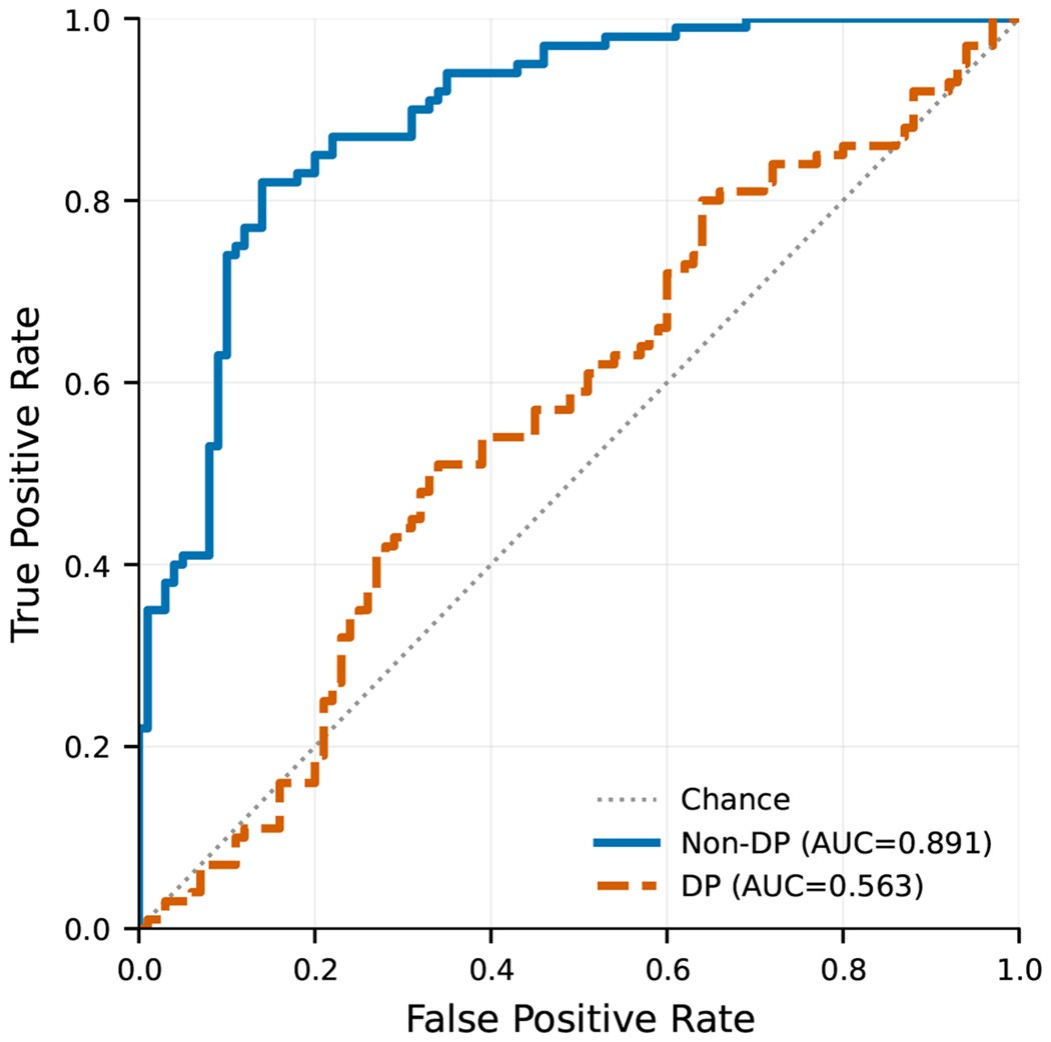
ROC curves for stage-level membership inference attacks (MIA) on CAMELYON17. The non-private baseline corresponds to NoisyFlow trained without differential privacy. It exhibits vulnerability to MIA with AUC = 0.891. In contrast, the DP-trained NoisyFlow model reduces the attack AUC to 0.563 compared with 0.891 for the non-private baseline, moving closer to chance-level performance.

Beyond utility, we evaluate whether differential privacy translates into empirical privacy protection using membership inference attacks (MIA) (Shokri *et al.* 2017). For each client, we partition the dataset into members (used during Stage 1 and Stage 2 training) and non-members (held out). After training, we compute per-example attack features, including flow-matching losses and, when applicable, OT-derived statistics, and train a lightweight binary MLP attacker to predict membership. We report attack accuracy and ROC AUC, where values near 0.5 indicate minimal membership signal. The non-private baseline is highly vulnerable, achieving an AUC of 0.891. In contrast, the DP-trained NoisyFlow model reduces the attack AUC to 0.563, approaching chance-level performance (Fig. 5). These results indicate that differential privacy substantially mitigates membership leakage in practice.

### 5.5 Cross-subject activity recognition: PAMAP2 wearables

Healthcare wearables continuously record physiological and behavioral signals such as heart rate and accelerometry. Models trained on data from a set of individuals often degrade when deployed on a new individual due to subject-specific physiology, sensor placement variability, and behavioral differences. This cross-subject problem is ubiquitous in digital health applications, where collecting labeled data from each new user is impractical.

We use the PAMAP2 Physical Activity Monitoring dataset (Protocol subset; Reiss and Stricker 2012), which contains synchronized streams from a heart-rate monitor and three inertial measurement units (IMUs) placed on the hand, chest, and ankle. Each time step is annotated with one of C=11 activity labels (walking, running, cycling, Nordic walking, ascending/descending stairs, vacuum cleaning, ironing, lying, sitting, standing). NoisyFlow operates on fixed-dimensional feature vectors rather than raw time series; we therefore segment each subject’s multivariate stream into fixed-length windows and represent each window as a single feature vector. Specifically, we downsample the raw 100 Hz stream by a factor of 4 (to 25 Hz), then form windows of 128 samples (5.12 s) with stride 64 (2.56 s). Each window is assigned the majority activity label, and we retain only windows with label purity ≥0.9. For each of the 10 sensor channels (heart rate plus 3-axis accelerometers from hand, chest, and ankle), we compute six summary statistics per window (mean, standard deviation, minimum, maximum, energy, and mean absolute derivative), yielding d=60 features per window.

We treat each source subject as a federated client with distribution $\mu_{i}$. Subject 107 is held out as the target domain with distribution ν, while subjects {101,102,105,106,108} serve as K=5 source clients. The target subject is split into a reference set ($n_{\text{ref-train}}=20$ windows) and a test set ($n_{\text{ref-test}}=400$ windows). Features are standardized using statistics computed on the union of all source samples and the target-reference split, with no leakage from the target test set.

Table 7 reports results. With only $n_{\text{ref-train}}=20$ labeled target windows, the Ref-train-only classifier achieves 75.3% accuracy. Training on raw windows $\tilde{x}$ without transport yields only 51.5% accuracy, confirming the substantial cross-subject distribution shift. After transport, $\tilde{y}$-only accuracy improves to 94.5%, a gain of 43.0 percentage points over the no-transport baseline. Combining 20 labeled target windows with transported windows $\tilde{y}$ yields 93.8% accuracy. The large improvement over Ref-train-only despite the small labeled target budget demonstrates NoisyFlow as a practical approach for cross-subject wearable adaptation.

**Table 7:** PAMAP2 cross-subject activity recognition (target subject 107).

Training data	*n*	Acc	Δ
Ref-train-only ($n_{\text{ref-train}}=20$)	20	0.753	—
$\tilde{y}$-only	200	0.945	+0.192
Ref-train + $\tilde{y}$ (20 + 200)	220	0.938	+0.185
Untransported $\tilde{x}$-only	200	0.515	−0.238

Δ : accuracy gain over Ref-train-only. Results are obtained with $\varepsilon =20.5,\delta =10^{-5},$ using Option C.

### 5.6 Computational efficiency

A key advantage of parameterizing the transport map as $T_{\theta }(x)=\nabla \Phi_{\theta }(x)$ is inference speed. Table 8 compares the time required to transport n=500 samples using our ICNN approach versus entropic OT (Sinkhorn; Cuturi 2013). The ICNN inference is approximately 13× faster (in ms) than solving the Sinkhorn iterations for each batch (with total speed up of 2×). This efficiency is crucial for the server-side synthesis step (Stage III), where the generator *u* and transport map $T_{\theta }$ must be evaluated repeatedly to construct the dataset ${\tilde{D}}_{\text{DA}}$.

**Table 8:** Inference time (ms) for transporting n=500 samples.

*d*	Sample	Transport (ICNN)	Transport (Sinkhorn)	Speedup (transport)	Speedup (total)
512	8.67	0.77	11.97	15.5×	2.19×
1024	8.32	0.89	11.46	12.9×	2.15×
2048	13.85	0.73	11.88	16.2×	1.76×
4096	9.33	0.91	11.87	13.0×	2.07×

Speedup compares the ICNN-based map $T_{\theta }$ against Sinkhorn. Both methods are executed on GPU; timings are measured on a single NVIDIA RTX 5090. For Sinkhorn, the regularization parameter is set to 1.0 with a maximum of 200 iterations.

### 5.7 Limitations and future work

Despite its promise, NoisyFlow assumes that source and target distributions can be meaningfully aligned via optimal transport; this assumption may break down under severe distribution shift or when feature supports overlap weakly. The current formulation further assumes access to a public reference distribution ν. If ν is small, noisy, or itself privacy-constrained, the target-side expectation in the OT objective is estimated with higher variance, degrading alignment quality without violating the source-side privacy guarantee. When ν is privacy-constrained, the data sharing protocol can be adjusted so that the target institution shares only DP trained model parameters rather than raw samples; extending the framework to formally account for a private ν (e.g. via DP-SGD on the target-side term) remains an important direction. On privacy interpretation, our strongest result (CAMELYON17, ε=0.32) corresponds to at most a 1.38× change in likelihood. The higher budgets in the single-cell and neurogenomics experiments reflect the small dataset sizes typical of biomedical studies; because DP-SGD utility scales with *n*, the trade-off is expected to improve as multi-institutional consortia grow.

## 6 Conclusion

NoisyFlow combines differential privacy with optimal transport to protect patient data while handling distribution differences across institutions. It aligns data distributions without exposing sensitive records, enabling secure cross-institutional collaboration with formal privacy guarantees. Our results show it is a promising approach for safer, more inclusive biomedical analysis across diverse data sources.

## Data Availability

The implementation of NoisyFlow and related dataset processing is available at https://github.com/gersteinlab/NoisyFlow.
